# Effects of urban airborne particulate matter exposure on the human upper respiratory tract microbiome: a systematic review

**DOI:** 10.1186/s12931-025-03179-9

**Published:** 2025-03-28

**Authors:** Sonia Arca-Lafuente, Beatriz Nuñez-Corcuera, Rebeca Ramis, Spyros Karakitsios, Denis Sarigiannis, Saúl García Dos Santos, Amanda Fernández-Rodríguez, Verónica Briz

**Affiliations:** 1 Viral Hepatitis Reference and Research Laboratory, National Center of Microbiology, Institute of Health Carlos III, Madrid, Spain; 2 Centro de Investigación Biomédica en Red en Enfermedades Infecciosas (CIBERINFEC), Instituto de Salud Carlos III (ISCIII), Madrid, Spain; 3 Air Pollution Area, National Center for Environmental Health. Institute of Health Carlos III (ISCIII), Madrid, Spain; 4 Cancer and Environmental Epidemiology Unit. Chronic Diseases Department, National Centre for Epidemiology, Institute of Health Carlos III (ISCIII), Madrid, Spain; 5 Centro de Investigación Biomédica en Red de Epidemiología y Salud Pública (CIBERESP), Instituto de Salud Carlos III, Madrid, Spain; 6 Laboratory of Environmental Engineering (Enve-Lab), Aristotle University of Thessaloniki, Department of Chemical Engineering, Thessaloniki, Greece; 7 National Hellenic Research Foundation, Athens, Greece

**Keywords:** PM_2.5_, PM_10_, Microbiome, Dysbiosis, Upper respiratory tract

## Abstract

Exposure to air pollutants has a direct impact on human health, resulting in increased mortality rates. Airborne particulate matter (PM) has major adverse effects on health and can be classified as high-risk respiratory particles (fine/PM_2.5_, aerodynamic diameter < 2.5 µm) or thoracic particles (coarse/PM_10_, aerodynamic diameter < 10 µm). In addition, airborne PM can carry microbial communities that alter the commensal microbiota and lead to dysbiosis. Our aim was to synthesize the current research evidence describing the association between air pollution exposure and the microbiome composition of the upper respiratory tract (URT) of the adult population. In this work, a systematic search of the PubMed, EMBASE and Scopus databases was conducted. A total of 9 studies published from 2018 to 2023 were included. 66.5% of the participants were exposed to PM_2.5_ concentrations higher than 40 µg/m^3^, and data showed that PM_2.5_ atmospheric levels were positively correlated with PM_10_ (r_s_ = 0.95, *p* < 0.001). All the reviewed studies performed 16S rRNA sequencing of the V3–V4 region from URT samples, using different methods. Overall, evidence of URT microbiome alterations after high PM exposure was observed, with seasonal and geographical influence. Discordant findings were found about bacterial diversity, with a predominant decrease after exposure to high PM levels. Regarding microbiome composition, the relative abundance of the Actinobacteria phylum declined following exposure to high levels of PM, but that of Bacteroidetes and Fusobacteria increased. The studies showed a low-middle risk of bias due to heterogeneity regarding sample processing, sequencing methods, and confounder control. To confirm the observed evidence of an association between PM levels and alterations in the URT microbiome, we strongly recommend that future research work be conducted in accordance with standard guidelines for reporting microbiome studies. In summary, the entry of fine and coarse particles into the URT is associated with microbial dysbiosis, increasing the risk of developing respiratory diseases and allergies.

*Prospero registration*: This systematic review was registered on PROSPERO (#CRD42023416230)

**Graphical Abstract:**

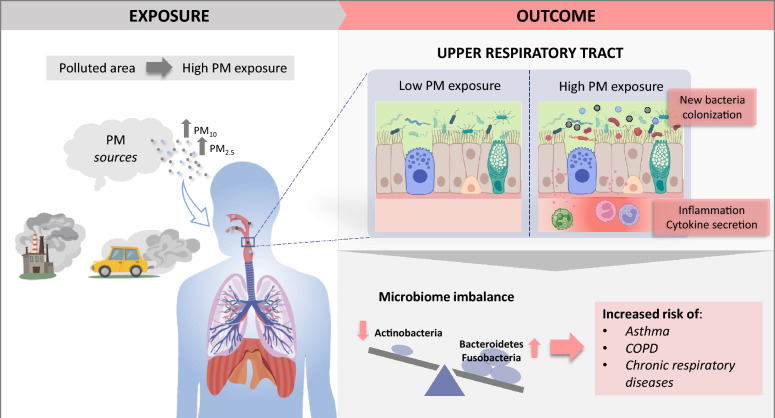

**Supplementary Information:**

The online version contains supplementary material available at 10.1186/s12931-025-03179-9.

## Introduction

Air pollution is considered a major public health concern, being the 13th cause of mortality worldwide, with 0.8 million deaths (1.4%) due to trachea, bronchus and lung cancer; cardiorespiratory pathologies; or respiratory infections [1]. The term air pollution refers to a combination of harmful substances such as particulate matter (PM), nitrogen oxides (NOx), ozone (O_3_), sulfur dioxide (SO_2_), carbonaceous aerosols, polycyclic aromatic hydrocarbons (PAHs), and heavy metals that are directly emitted from primary sources or formed by secondary photochemical reactions. Among them, the component with the greatest adverse effects on human health is PM, a complex mixture of chemicals, mineral dust, microorganisms and other organic substances [2].

PM is classified according to its equivalent aerodynamic diameter as thoracic particles (coarse/PM_10_) with diameters < 10 µm [3], and high-risk respiratory particles, including ultrafine particles (UFP), with diameters < 0.1 µm, and fine particles (PM_2.5_), with diameters < 2.5 µm. Both, PM_10_ and PM_2.5,_ are ingested and deposited in the upper airway. Airborne PM acts as a transmission vehicle for potential microbial pathogens into the respiratory system [4, 5]. Microbiome communities vary with body location, and changes in their composition are directly related to the development of several pathologies.

The upper respiratory tract (URT) is the main entry way of environmental pollution in the human body. PM_2.5_ owing to its size, can penetrate deep into the lower respiratory tract (LRT). But as it passes through the airways, exposure to PM_2.5_ can induce epithelial alterations, thus promoting inflammation and microbial dysbiosis, increasing the risk of suffering URT’s affections with symptoms such as runny nose, or cough [6, 7]. The alteration of the epithelium bound to the carriage of microorganisms by PM_2.5_, increases the susceptibility of suffering respiratory infections [8, 9]. The colonization of the habitat of healthy bacterial communities can induce immune responses via Th17 cell activation, which leads to inflammation and alteration of the bacterial composition of the URT [10, 11]. The inflammation triggered after the exposure to high levels of either fine or coarse particles is associated with an increase of inflammatory respiratory disorders of the nasal cavity, like allergic rhinitis and chronic rhinosinusitis [12–14]. In addition, once PM_2.5_ and UFP cross the URT, they can reach the lower lung tract and travel through the alveoli to the bloodstream [15]. Besides, the particles, specially PM_2.5_, can be brushed up from the lungs by the mucociliary system and reach the intestines [16], with the consequent shift on the intestinal microbiome [17]. As a consequence, PM exposure is especially related not only to the development of both respiratory and cardiovascular diseases [18], but also to additional gastrointestinal inflammatory disorders such as inflammatory bowel disease (IBD), colorectal cancer, or appendicitis [2, 19, 20], as well as other organ affections such as brain damage [21], and chronic kidney and liver [22, 23] diseases.

Owing to the attribution of PM to all-cause mortality, systematically reviewed for long-term exposure (defined as months to years) [24] and short-term exposure (as days to four weeks) [25, 26], in 2021, the WHO established more restrictive recommendations for annual and daily values: 5 µg/m^3^ for PM_2.5_ and 15 µg/m^3^ for PM_10_ and a maximum daily exposure of 15 µg/m^3^ for PM_2.5_ and 45 µg/m^3^ for PM_10_ [27]. On the basis of those recommendations, countries should either establish their own regulations or, within the European Union, follow the EU Directive. As shown in Table 1, annual limits differ among some countries from 5 to 25 µg/m^3^ for PM_2.5_ or 15–40 µg/m^3^ for PM_10_, and daily limits vary in the range of 15–35 µg/m^3^ for PM_2.5_ or 45–150 µg/m^3^ for PM_10_.

**Table 1:** Limit values for PM_2.5_ and PM_10_ daily and annual exposure

Region		Regulation	Limit values	PM_2.5_ (µg/m^3^)	PM_10_ (µg/m^3^)	
Worldwide		WHO Guidelines, 2005	Daily	25	50	
			Annual	10	20	
Worldwide		WHO Guidelines, 2021	Daily	15	45	
			Annual	5	15	
European Union		Directive 2008/50/CE, Ambient Air Quality and cleaner air for Europe	Daily		50	
			Annual	25	40	
United States of America		National Ambient Air Quality Standard for PM_2.5_ and PM_10_ (Enviromental Protection Agency)	Daily	35	150	
			Annual	15		
China		Ambient Air Quality Standard, GB 3095-2012	Daily	35^a^/75^b^	50^a^/150^b^	
			Annual	15^a^/35^b^	40^a^/70^b^	
Overall			Daily	15–75	45–50	
			Annual	5–20^a^/35^b^	15–40^a^/70^b^	

Note: PM_2.5_ and PM_10_ values recommended by the WHO and established by environmental agencies and governments of the European Union, the United States of America and China

^a^Urban areas

^b^Rural areas

In the absence of systematic knowledge on the special role of high-risk PM, we aimed to conduct the first systematic review investigating the effects of exposure to elevated PM levels on the upper respiratory tract microbiome and its potential associated health consequences.

## Methods

### Protocol and registration

This systematic review was performed according to the Preferred Reporting Items for Systematic Reviews and Meta-Analysis of Diagnostic Test Accuracy (PRISMA-DTA) guidelines and registered on PROSPERO (#CRD42023416230).

### Eligibility criteria

The research question was developed via the PICO structure (Participants, Interventions/Exposures, Comparisons and Outcomes). The PICO question of this review was “How does airborne particulate matter exposure impact the composition of the human upper respiratory tract microbiome?”. The inclusion criteria were as follows: (1) randomized clinical studies and/or observational studies; (2) studies that performed 16S rRNA high throughput sequencing to determine the relative abundance of microbial phyla from nasopharyngeal or oropharyngeal samples; (3) studies including adults older than 18 years; and (4) studies assessing PM_2.5_ and/or PM_10_ exposure. The exclusion criteria were as follows: (1) studies without available 16S rRNA raw sequencing data or microbial abundance values; (2) microbiome studies on non-URT locations/tissues; (3) studies without quantitative published data on the PM exposure concentration; and (4) studies with data published in abstracts only or presented as slides, posters, or letters.

### Information sources and search strategy

Studies were identified by conducting a systematic search through Medline/PubMed, EMBASE and Scopus for research articles published until September 2024. The search strategy used keywords, controlled vocabulary, and Boolean operators to describe each intervention and outcome of interest. The search was conducted through articles published in PubMed, EMBASE, and Scopus, with the search terms “(‘shot-gun’ OR ‘16s’ OR “RNA, Ribosomal, 16S” [MeSH Terms] OR ‘sequenc*’ OR ‘diversity’ OR ‘richness’ OR ‘abundance’) AND (‘microbiome’ OR ‘microbi*’ OR “microbiota” [MeSH Terms]) AND (‘upper respiratory’ OR ‘pharyngeal’ OR ‘nasal’ OR ‘orop*’ OR ‘oral’ OR ‘buccal’) AND (((“particulate matter”[MeSH Terms] OR (‘particulate’ AND ‘matter’) OR ‘particulate matter’) AND (‘2.5’ OR ‘10’)) OR ‘pm2.5’ OR ‘pm10’ OR ‘air pollution’ OR ‘airborne particle’)”. The records obtained were screened on the basis of title and abstract. Eventually, full-text articles were assessed for admissibility based on the eligibility inclusion and exclusion criteria previously established.

### Data extraction

Data extraction from the included papers was performed and independently cross-checked by two investigators (S.A.L. and R.R.). The reference limit values for the permissible ambient concentration of PM were compiled by two researchers according to current Ambient Air Quality regulations (B.N.C. and S.G.D.S.). When the data were unclear or in doubt, other researchers (A.F.R. and V.B.) were consulted to reach a consensus. In an attempt to include the majority of studies and to complete any incomplete or missing data, the authors of individual studies were contacted by S.A.L. up to three times. Studies that fulfilled the inclusion criteria but whose results were not provided after three attempts to contact the study authors were excluded. When more than one paper studying the same cohort was found, only the study with the most extensive cohort was included, excluding the remaining overlapping studies or data. Data were recorded and managed via Excel spreadsheets, references were managed with EndNote software, and data analysis and visualization were performed with RStudio.

### Outcomes and prioritization

This review evaluated the changes in the microbial phyla composition of the URT (oro- or naso-pharyngeal samples) associated with high exposure to PM_2.5_ and/or PM_10_ in adult subjects.

In accordance with WHO recommendations and considering the highest admitted levels for high-risk respiratory particles in urban settings worldwide, in this study, the established cutoff for PM_2.5_ exposure was 40 µg/m^3^, and population groups were classified as ‘low exposure’ ([PM_2.5_] < 40 µg/m^3^) or ‘high exposure’ ([PM_2.5_] > 40 µg/m^3^). Considering previous evidence of a positive correlation between PM_2.5_ and PM_10_ atmospheric concentration [28, 29] and the strong Spearman correlation obtained with our data (see "Characteristics of the studies included in the systematic review" section), we assume that the stablished cutoff would also let us accurately classify the studies according to PM_10_ exposure levels.

### Risk of bias in individual studies

The quality of the selected studies was estimated based on the Collaboration for Environmental Evidence Critical Appraisal Tool (CEECAT), which was developed for the assessment of the effectiveness of interventions or impacts of exposures in environmental management. CEECAT consists of a question checklist divided into 7 criteria: risk of (1) confounding biases, (2) postintervention/exposure selection biases, (3) misclassified comparison biases, (4) performance biases, (5) detection biases, (6) outcome reporting biases, and (7) outcome assessment biases. The overall risk of bias was classified as follows: (i) low risk when all 7 individual criteria had a low risk of bias; (ii) medium risk if at least one criterion had a medium risk of bias; and (iii) high risk if at least one criterion was classified as having a high risk of bias.

To assess the quality of the reporting of metagenomic studies, the “Strengthening the Organization and Reporting of Microbiome Studies” (STORMS) checklist (version 1.03) was used.

### Data

Processed data were extracted from the manuscript or supplementary material of each individual study, which included the phylum relative abundances of the bacterial community, family and genus principal findings, alpha diversity values, beta diversity values and any additional outcomes when available. When a study investigated multiple exposure subgroups within a single PM exposure category within this systematic review, the relative abundances of that study were estimated as the geometric mean of the reported values.

When available, 16S rRNA sequencing raw data from control subjects (those with no reported medical condition or antibiotic use in the previous month) were extracted from public repositories and reanalyzed to obtain relative abundance values. Downstream analyses were carried out with the online platform Galaxy. Pair-end sequences were trimmed at the start of each read by 21 nt to eliminate primer sequences, and forward/reverse reads were truncated with an overlap of 30–40 nt. Subsequent filtering was performed via the statistical software R (v 4.2.2) (www.r-project.org), following DADA2 pipeline (v 1.30.0) [30]. Taxonomic assignment was carried out through the GreenGenes (v 13.8) database. The data filtering parameters were set to retain OTUs that appear more than twice. Subsequently, read counts were normalized within each sample using median sequencing depth, before estimating relative abundances, using the phyloseq package (v 1.46.0) [31]. Finally, relative abundance values were obtained. Either the collected or recalculated relative abundance data are displayed as bar plots and forest plot diagrams.

## Results

### Study selection

**Fig. 1:**
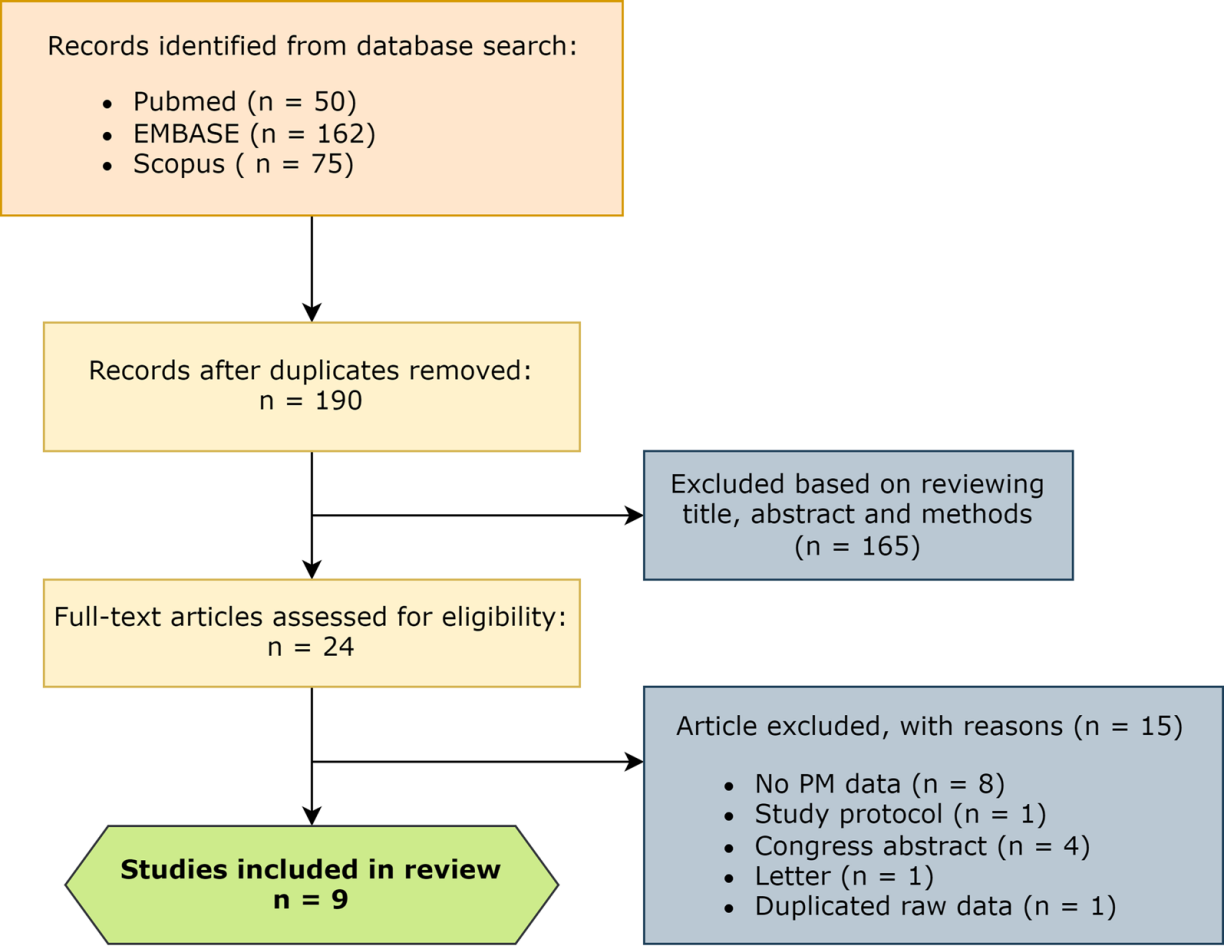
PRISMA flowchart showing the screening process for the inclusion of selected studies

The initial literature search revealed 355 eligible studies. After duplicate removal, 225 remaining studies were screened on the basis of article title, abstract and methodology. A total of 24 studies were selected for full-text eligibility, 15 of which were excluded for different reasons: not providing PM data (n = 8), a study protocol yet to be conducted (n = 1), a congress abstract (n = 4), a letter (n = 1), or a description of different outcomes from the same data of an already included study (n = 1). A summary of the literature screening process is summarized in a PRISMA flowchart (Preferred Reporting Items for Systematic Reviews and Meta-Analyses, Fig. 1).

### Characteristics of the studies included in the systematic review

A summary of the characteristics of all included studies, including participant demographics and PM sampling methods, is provided in Table 2. Briefly, the nine studies were published between 2018 and 2023, two of which were conducted in Italy and seven in China. The total number of participants was 486 (66.5% belonging to the ‘high exposure’ group), and all were exposed to real environmental conditions, which include both PM_2.5_ and PM_10_. As presented in Table 3, all studies reported PM_2.5_ and PM_10_ levels of exposure except two [10, 32] that reported only PM_2.5_ levels. When both data were available, PM_10_ concentration was positively associated with PM_2.5_ levels (r_s_ = 0.95, *p* < 0.001). Based on this correlation and considering fine particles’ concentration as an indicator of coarse particles’ [33], we decided to set the exposure cutoff based on PM_2.5_ to guarantee availability of quantitative data for all the nine studies (cutoff of 40 µg/m^3^).

**Table 2:** Characteristics of the population and PM sampling methods of each included study

Study	Mariani et al. [37]	Mariani et al. [14]	Lin et al. [38]	Li et al. [39]	Qin et al. [34]	Zhao et al. [32]	Zhao et al. [10]	Du et al. [36]	Li et al. [26]
Study population	Adult subjects	Allergic rhinitis and Healthy aldult subjects	Adult subjects	Adult subjects	Adult subjects	Adult subjects	Adult subjects with asthma	Adult subjects	Adult subjects
Country	Italy	Italy	China	China	China	China	China	China	China
PM Exposure Group	Low	Low	Low	High	High	Low/High	High	Low/High	High
PM sampling duration	7 days	7 days	2 months	2 years	3 days	30 days	30 days	2 months	3 months
Sampling season	Winter	Spring	Summer/Fall	Winter	Winter	Spring	Winter	Fall	Fall
Exposure assesment	(1) Personal cascade impactor sampler (2) Chemical Transport Model (3) fixed monitoring stations	Regional Environmental Protection Agency (ARPA Lombardy)	GRIMM Aerosol Technik Ainring (located in a roofed building within 200 m of participants’ dormitories)	Thermo5030 SHARP	Official air quality forecast	Open Environment Data Center of China	Open Environment Data Centre of China	Online platform for air quality monitoring (aqistudy)	Tisch Environmental TE-6070 (located on the rooftop of campus building)
Swab location	Nasal	Nasal	Oral	Throat	Nasopharyngeal	Nasopharyngeal	Oropharyngeal	Oral (wash)	Nasal (wash)
Research design	Cross-sectional	Cross-sectional	Longitudinal	Cross-sectional	Longitudinal	Longitudinal	Cross-sectional	Longitudinal	Longitudinal
Female	23 (57.5%)	24 (48.0%)	30 (75.0%)	61 (53.5%)	44 (53.0%)	3 (37.5%)	16 (72.0%)	11 (52.38%)	48 (70.6%)
Age (SD)	48.6 ± 8.4	41.0 ± 14.6	24.0 ± 1.5	19.9 ± 0.4	39.2 ± 11.5	21.9 ± 0.35	53.9 ± 9.6	26.8 ± 6.7	18.62 ± 0.71

Note: ‘PM Exposure group’ indicates the classification established in the present systematic review. The values are expressed as absolute numbers (percentages) or as the means ± standard deviations

**Table 3:** Summary data of air pollutants and weather conditions described in the included studies

Study	Exposure Subgroup within the individual study	PM_2.5_ (µg/m^3^)	PM_10_ (µg/m^3^)	NO_2_ (µg/m^3^)	SO_2_ (µg/m^3^)	O_3_ (µg/m^3^)	Temperature (^°^C)	Humidity (%)
Mariani et al. [37]		36.5	48.3					
Mariani et al. [14]	Healthy subjects (HS)	14.9	22.9					
	Allergic rinitis (AR)							
Lin et al. [38]	Low	20.3 ± 6.5	45.9 ± 24.5	23.7 ± 13.7	7.7 ± 2.4	71.9 ± 41.8	25.5 ± 5.9	68.4 ± 15.1
	High	59.1 ± 8.1						
Li et al. [39]	Region A	72.5	100	N.A	N.A	N.A		
	Region B	72.5	100	N.A	N.A	N.A		
	Region C	92.5	135	N.A	N.A	N.A		
Qin et al. [34]	Pre-smog	80–128	150					
	Post-smog	217–287	314–416					
Zhao et al. [32]	S, M	101.0 (0.9)		41.1 (4.5)	14.2 (1.0)	93.3 (22.4)	8.9 (1.0)	64.2 (2.6)
	S, D7	85.1 (2.7)		41.1 (11.3)	14.0 (3.7)	117.7 (29.6)	10.9 (0.9)	62.7 (11.7)
	S, D0	110 (12)		46 (21)	8 (12)	52 (152)	6.5 (10.5)	77 (18)
	F, M	32.7 (2.3)		45.2 (0.8)	12.2 (0.8)	165.2 (21.1)	22.4 (0.4)	63.6 (0.2)
	F, D7	28.7 (1.1)		45.2 (0.8)	10.6 (3.2)	92.4 (27.8)	20.6 (1.1)	83.6 (15.4)
	F, D0	22 (19.5)		36 (11.25)	13 (2.25)	155 (4.5)	23.0 (2.6)	64 (12)
Zhao et al. [10]	W1, M	100.9 ± 12.6		48.1 ± 8.0	19.8 ± 3.1	66.3 ± 14.8	4.7 ± 3.6	53.0 ± 5.5
	W1, D7	95.1 ± 34.3		49.4 ± 8.1	18.4 ± 5.3	82.8 ± 47.4	5.7 ± 4.7	56.6 ± 15.4
	W1, D0	73.2 ± 30.7		51.8 ± 20.2	18.0 ± 5.2	91.9 ± 58.9	9.2 ± 7.8	55.7 ± 19.8
	W2, M	109.7 ± 6.5		63.8 ± 3.1	18.1 ± 1.4	45.8 ± 1.7	3.2 ± 2.4	54.5 ± 4.6
	W2, D7	110.5 ± 31.4		68.0 ± 7.7	18.2 ± 3.5	45.3 ± 9.5	2.0 ± 2.3	52.0 ± 6.1
	W2, D0	97.0 ± 51.0		74.4 ± 17.8	21.5 ± 5.5	45.3 ± 23.0	3.5 ± 3.2	46.0 ± 10.2
Du et al. [36]	D0	5 (6)	32 (15)				11.2 (2.9)	27 (12)
	D30	90 (36)	213 (159)				3.8 (2.4)	51 (37)
	D60	21 (23)	59 (37)				−4.8 (3.6)	26 (13.8)
Li et al. [26]	S1	222.6 ± 111.9	134.4 ± 59.0	54.6 ± 29.0	29.1 ± 13.7	101.2 ± 72.2	26.6 ± 2.9	57.5 ± 15.6
	S2	99.1 ± 67.8	111.4 ± 68.5	52.7 ± 23.4	45.8 ± 17.8	23.7 ± 24.2	5.8 ± 3.6	36.8 ± 15.1

N.A: recorded data not publicly available. Values are reported as absolute numbers (percentages), means ± standard deviations, or medians (interquartile ranges)

Two studies analyzed microbiome alterations after a high-pollution episode, but PM_2.5_ levels both before and after the high-pollution episode were above 40 µg/m^3^ [34, 35]. In both longitudinal studies, same individuals at different time points were involved. Although Zhao et al. [10] performed their study during the winter season for two consecutive years, the individuals involved were different, defining their study as cross-sectional. Further data on the air pollutants and weather conditions measured in each study are included in Table 3.

Most of these studies reported microbiome composition in subjects with no described respiratory disorders, but two of them analyzed the impact of PM exposure on allergic rhinitis (AR) patients [14] and patients with asthma [10]. Finally, one study investigated the effect of azithromycin treatment on microbiota resilience to ambient pollution [10, 36].

All PM measurements were recorded in urban locations. However, Mariani et al. [37] assessed PM levels with a combination of an urban monitoring station and personal sampling devices, so we cannot confirm that all the measurements in their study came from a city area [37].

### Microbiome analysis methods

Microbiome sampling was performed from the URT in all the studies via different methods (Table 2). To reduce heterogeneity, only oral wash data reported by Du et al. [36] were selected for analysis, and sputum samples were discarded. The nasal, oral, nasopharyngeal and oropharyngeal microbiomes are considered equivalent habitats for the study of the upper respiratory microbiome.

As shown in the STORMS metagenomic checklist (Additional file 1), all studies included an adequate description of the study design and participants. The time of storage before DNA extraction was not reported in any of the studies, which is a potential bias for DNA integrity. All the studies amplified the 16S rRNA V3-V4 region using different primers, and all the reported sequencing methods used were conducted on the Illumina HiSeq platform [34], Illumina NovaSeq 6000 platform [38], Ion S5™ XL platform [35], and Illumina MiSeq platform in the rest of studies. Raw sequencing data in public databases were only available for two studies [14, 36]. For the remaining studies, raw sequencing data were not provided after three contact attempts. A summary of the methods used for DNA extraction, sequencing, bioinformatic and statistical analysis is collected in Additional file 2.

Alpha-diversity was analyzed in all studies by the Chao 1, Simpson and Shannon indices, except one [36], who reported only the Shannon index. Additionally, the ACE index [10, 32, 34, 38, 39], whole-tree phylogenetic diversity [14, 34, 37], equitability [35], and observed richness [14, 34, 36] were used to display the results. However, one study did not publish any diversity index results [39].

### Analysis of microbiome alterations due to particulate matter exposure

The principal findings reported by the reviewed studies are summarized in Table 4. In the following sections, we specify the most relevant data regarding microbial diversity and composition.

**Table 4:** Summary of the principal findings of the reviewed studies

Study	Phylum	Family	Genus/order	Alpha- and beta-diversity	Additional outcomes
Mariani et al. [37]	Representation: Actinobacteria > Proteobacteria > Firmicutes Actinobacteria showed an inverse association with PM	Representation: Corynebacteriaceae > Moraxellaceae and Staphylococcaceae Moraxellaceae was the only family that showed a positive association with PM	*Moraxella* genus presented a positive association with PM levels	Regression models revealed alpha-diversity indices reduced after high PM exposure	
Mariani et al. [14]	Representation: Actinobacteria > Proteobacteria > Firmicutes		In healthy subjects, *Corynebacterium* and *Staphylococcus* abundances were higher	Observed reduction in microbiota diversity in **AR** patients, probably linked to their disease. Less variations after PM exposure in **AR** than in **HS**, probably due to a dysfunctional microbiota. Intragroup distance was higher in AR patients. However, no significance differences were identified between the groups	Higher plasmatic extracellular vesicles (EVs) release by bacteria probably linked to the pro-inflammatory activation triggered by PM exposure
Lin et al. [38]			After high exposure, enrichment in order *Bacteroidales* and lower relative abundance of order *Bacillales*	Lower microbiota diversity after short-term exposure to high levels of PM_2.5_. However, no significat differences were observed within groups	PM was significantly associated with higher inflammatory response, elevated stress and decreases oral immunity
Li et al. [39]	Representation: Bacteroidetes, Firmicutes, Proteobacteria, Actinobacteria, and Fusobacteria Bacteroidetes relative abundance decreased in 50% in region **C**. Actinobacteria increased almost 100% in **C** compared to **A**. Firmicutes abundance was significantly higher in the **B** and **C** regions	Prevotellaceae, Veillonellaceae, Porphyromonadaceae, Fusobacteriaceae Paraprevollaceae and Flavobacteriaceae reduced in region **C**. Lachnospiraceae and Ruminococcaceae increased in region** C**	*Prevotella*, *Veillonella*, *Fusobacterium*, *Camphylobacter* and *Capnocytophaga* were negatively associated with pollution. Genera *Prevotella*, *Porphyromonas*, *Peptostreptococcus* and *Moraxella* were higher in region **A** and *Rothia* in region **B**	High PM exposure increased microbiota diversity in the oropharynx	
Qin et al. [34]	Representation: Proteobacteria > Firmicutes > Bacteroidetes > Fusobacteria > Actinobacteria. The read numbers of Firmicutes, Fusobacteria, and Actinobacteria were increased in the post-smog samples	Post-smog were enriched in Veillonellaceae	35 new genera were found in post-smog, enriched in *Leptotrichia*, *Streptococcus*, *Haemophilus*, *Moraxella*, and *Staphylococcus* (300% higher), and reduced in *Prevotella* and *Neisseria*	Alpha diversity indices were higher in post-smog (high PM exposure increases diversity), although evenness indices (Shannon and Simpson) differences did not reach significance	Wearing a mask and smoking had a significance influence on microbiota. Wearing a mask was negatively correlated (although not significant) with richness. No differences observed between pre- and post-smog in people wearing masks
Zhao et al. [32]	Overall representation: Firmicutes > Proteobacteria > Bacteroidetes Acidobacteria and Gemmatimonadates were positivly correlated with PM_2.5_ exposure		Overall enriched in *Streptococcus, Neisseria, Haemophilus, Porphyromonas, Prevotella,* and* Fusobacterium* *Symbiobacterium* was positively associated with PM_2.5_ *Streptococcus* and *Prevotella* were positively associated with temperature	Alpha-diversity: no variation between groups Beta-diversity differed among groups, reflecting composition changes of the nasopharyngeal microbiota after increased PM_2.5_ exposure	PM_2.5_ and temperature are the most important environmental factors that alter composition and community of nasopharynx microbiota, increasing colonization of bacteria from soil
Zhao et al. [10]	Representation: **W1**: Firmicutes > Proteobacteria > Bacteroidetes > Fusobacteria > Actinobacteria > Thermi **W2**: Proteobacteria > Actinobacteria > Firmicutes > Bacteroidetes > Fusobacteria > Thermi		**W1**: enriched in *Streptococcus*, *Prevotella*, *Haemophilus* and *Actinobacillus* **W2**: enriched in *Rothia*, *Thermus*, *Actinomyces*, *Fusobacterium*, *Cupriavidus*, *Leptotrichia* and *Acinetobacter*. *Staphylococcus* and *Moraxella* were extremely low in both	**W1** had a lower bacterial diversity than **W2**. Additionally, communities’ representation differed between **W1** and **W2,** showing that higher environmental PM_2.5_ exposure increased the colonization of bacteria from environmental soil in the oropharynx	NO_2_ was the most important factor with significant effects on the composition and community structure of the oropharyngeal microbiome, especially in people with asthma, followed by PM_2.5_ and O_3_
Du et al. [36]			*Lachnoanaerobaculum, Leptotrichia, Prevotella, and Veillonella* were the most represented genera, independently of ambient PM_2.5_ levels	No variations in alpha-diversity due to PM_2.5_ exposure	No variations in the airway microbiota of study subjects due to higher PM_2.5_ exposure. Patients under azithromycin treatment are more susceptible to microbial alterations after high pollution episodes, better reflected in the sputum fraction
Li et al. [26]		Bacillales, Staphylococcaceae, and Staphylococcus were associated with high PM_2.5_ exposure, while Clostridia or Moraxellaceae representation was higher under low exposure		Alpha-diversity indices were negatively associated with PM_2.5_ levels	High PM_2.5_ exposure was associated with acute increase in inflammatory and oxidative stress biomarkers

#### Microbial diversity

Discordant findings are described throughout the studies in relation with microbial diversity. First, three studies described a significant increase in bacterial diversity after exposure to high PM levels, reaching significance levels in two of them (richness-based metrics: *p* < 0.001 [34] and *p* = 0.005 [10]; phylogenetic diversity: <0.05 [34]). In contrast, three studies indicated that exposure to high levels of PM translated into a reduction in alpha diversity indices (richness-based metrics: *p* < 0.01 [35, 38], *p* < 0.25 [37]; phylogenetic diversity: *p* < 0.01 [37]).

Of note, Mariani et al. [14] compared the variations in bacterial diversity among allergic rhinitis patients (AR) and healthy subjects (HS) and reported a greater intragroup distance distribution in AR patients, probably due to their disease condition. This led to a positive association between PM_2.5_ levels and richness and evenness indices only in AR patients (*p* = 0.01 and *p* = 0.04), while alpha-diversity indices decreased in HS (*p* = 0.03 and *p* = 0.04).

No statistically significant differences in alpha-diversity were reported in the rest of studies. An illustration of the general trends in alpha-diversity is included in Additional File 3.

#### Microbiome composition

Taxonomy was heterogeneously reported, as most of the studies revealed different outcomes linked to PM exposure, whereas two of them [35, 38] did not report any abundance data. Only two studies have publicly available 16S rRNA raw sequencing data (BioProject accession numbers PRJNA646474 [14] and PRJNA565553 [36]). Raw sequencing data of control subjects from the abovementioned projects were analyzed as previously described to achieve greater homogeneity of the microbiome composition results.

**Fig. 2:**
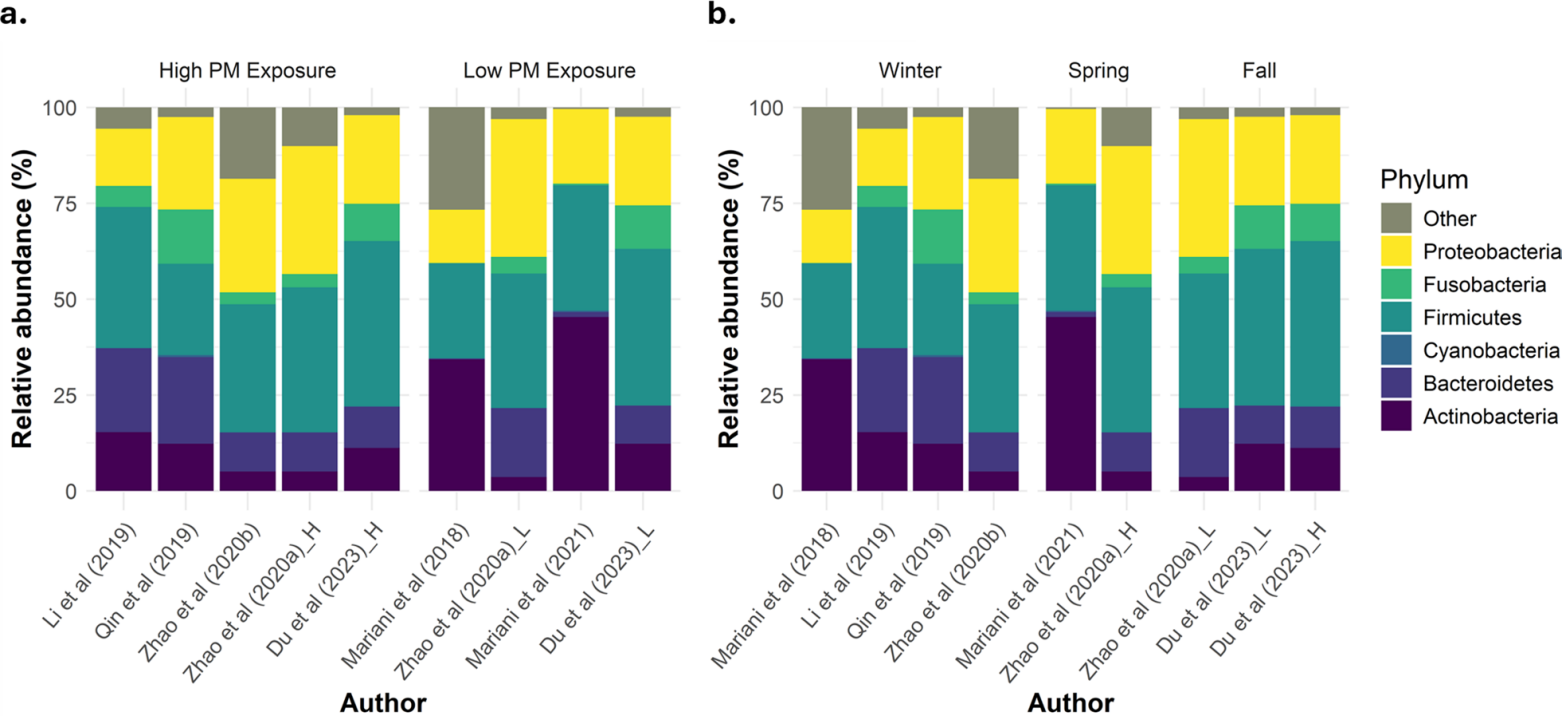
Differences in the distribution of the upper respiratory microbiome composition at the phylum level by PM exposure (*left panel*** a**) or by season (*right panel* **b**), where “Zhao H. (2020a)” is a study in healthy adults [32], and “Zhao H. (2020b)” the study on adults with asthma [10], subdivided into low (L) and high (H) subgroups. The data are presented as the relative abundances (%) observed for each phylum

**Fig. 3:**
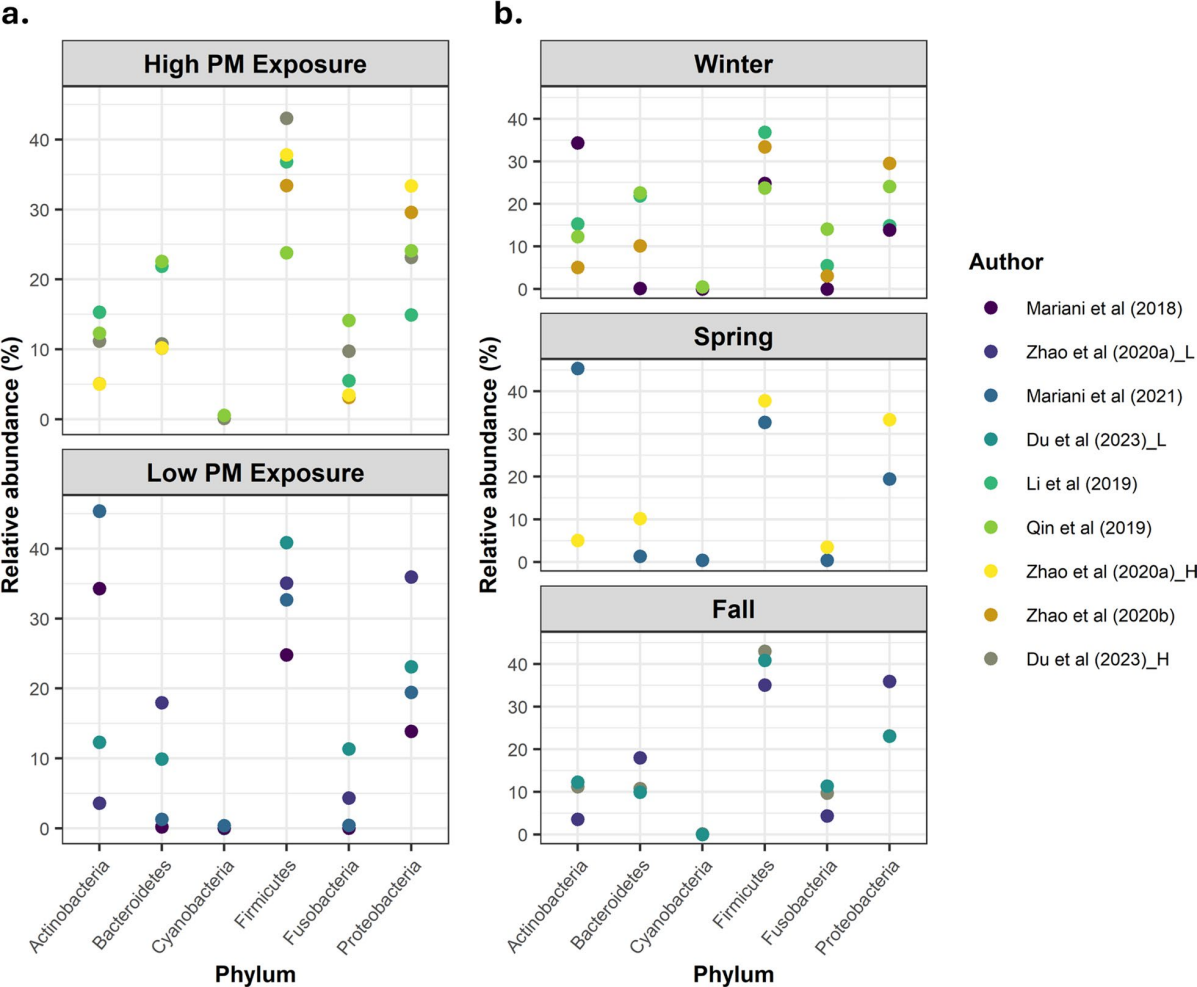
Differences in the distribution of the upper respiratory microbiome composition by phylum among the high- and low-exposure groups (*left panel* **a**) or among the sampling seasons (winter, spring, and fall, *right panel*** b**). The data are presented as the relative abundances (%) estimated from raw data [14, 36] or reported by the individual studies (rest), for each phylum

The available data for microbiome composition at the phylum level are summarized in Fig. 2, and those at the phylum level are shown in Fig. 3. Additionally, the microbiome composition gather by the available PM_10_ data is included in the Additional file 4. First, relative abundances were compared by PM exposure level. In the low-exposure group, Firmicutes (24.8–40.9%) and Proteobacteria (13.9–35.9%) were the dominant phyla. Actinobacteria (3.6–45.3%) presented the greatest variability among the low-exposure group, representing the main phylum among the Italian population, as depicted in Fig. 2A (studies conducted by Mariani et al. and Additional file 5. The abundances of Bacteroidetes (0.2–18.0%) and Fusobacteria (0.03–11.3%) also varied, whereas those of Cyanobacteria (0.01–0.4%) were almost irrelevant.

In contrast, the abundance of Bacteroidetes was greater in the high-exposed population (10.2–22.6%). Although the results of Zhao et al. [10] represented the lowest scenario for Bacteroidetes, the authors reported an increase in this phylum after high PM exposure. The most represented phyla within the high-exposure studies were Firmicutes (23.8–43.1%) and Proteobacteria (14.9–33.4%). Actinobacteria (5.0–15.3%) and Fusobacteria (3.1–14.1%) were equally represented in the URT of individuals exposed to high levels of PM. Again, Cyanobacteria (0.1–0.6%) presented the lowest representation. Notably, no data were available in some of the studies concerning Cyanobacteria and Fusobacteria, probably because of the insignificant abundances obtained. Firmicutes was the phylum with less variance observed among the low- and high-exposure groups (Fig. 3A).

Secondly, we noticed that the time of year when samples were collected varied among the reviewed studies. To consider this factor, the data were grouped by season. As shown in Fig. 3B, during the fall season, a slight increase in Firmicutes and Fusobacteria was observed compared with those in the winter and spring; Firmicutes was the first most abundant phyla in the URT in fall, whereas the abundance of Actinobacteria was lower in this season. The abundance of Proteobacteria was also slightly greater during fall. In contrast, during spring, Fusobacteria and Bacteroidetes were underrepresented compared with those in winter/fall. With respect to winter, greater variations in relative abundances were observed for all phyla, without a clear trend. None of the studies determined microbiome composition at the phylum level during the summer.

Additionally, since studies were performed either in Italy or China, a comparison of the composition of the URT microbiome between both countries showed that Actinobacteria and Bacteroidetes were differently distributed depending on location (Additional file 5). Specifically, Actinobacteria relative abundance was 2–3 times higher in Italy than China, while Bacteroidetes abundance was reduced. These findings indicate that location may affect the interpretation of the effects of PM on those phyla.

Only four studies analyzed the microbiome composition at the family level, with discrepant results. Some authors have reported significantly positive associations of Moraxellaceae (*p* < 0.05) [37] and Veillonellaceae (LDA score > 4.0) [34] with the PM concentration, while others reported a significantly negative association for Veillonellaceae (*p* < 0.05) [39]. In line with these findings, most of the studies reported that the *Moraxella* genus abundance presented a positive association with PM levels [34, 37, 39], except two that reported a decrease in *Moraxella* genus and Moraxellaceae family associated with PM_2.5_ [10, 35]. With respect to other genera, each study detailed different variations, which are summarized in Table 3.

### Quality appraisal

The quality of the selected studies was analyzed via the CEECAT tool. The detailed quality evaluation can be found in Additional file 6. Briefly, the CEECAT tool revealed that all studies had a low risk of bias in terms of study group representation, statistical analysis, exposure assessment methods, and outcome assessment and reporting. It was unclear if the data analysts were aware of the exposure level of each study group or if a blind analysis of the data was performed, except for Du et al. [36], who clearly stated that the entire study was double-blind. Therefore, a medium risk of bias was considered in the remaining studies. Multiple air pollutants (SO_2_, NO_2_, CO, and O_3_) as well as weather conditions (temperature and humidity) are considered potential confounding factors. The mentioned confounders were not examined in three studies [14, 34, 37], indicating a high risk of introducing bias in the study of the outcome effects.

As recorded in Table 2, the exposure assessment method varied among all the studies. This leads to distinct uncertainty levels related to the PM sampler used, which was considered a limitation in pollution data comparisons.

#### Covariate data relevant to the respiratory microbiota

##### Lifestyle behaviors

Most of the studies considered patients’ smoking habits (n = 8/9) [10, 32, 34–39], and five excluded patients from the microbiome study because of smoking (n = 5/9) [10, 35, 36, 38, 39]. Additional lifestyle factors, such as drinking [38], taking supplements [38], or illicit drug use were considered in individual studies [14, 37].

Antibiotic treatment for a month was recorded in six studies (n = 6/9) [10, 32, 34–36, 39], and only four of them excluded subjects because of antibiotic use [10, 32, 35, 39]. Qin et al. [34] did not exclude subjects with antibiotic use but determined that this factor did not have significant effects on the microbiome composition after exposure to high PM levels. Du et al. [36] specifically studied the effects of antibiotic use on the microbiome, as well as the recovery of the upper respiratory microbiome one month after treatment.

##### Comorbidities and medical conditions

Different health conditions were considered among the studies. The most common exclusion criteria were cardiovascular and respiratory diseases such as asthma, chronic obstructive pulmonary disease** (**COPD) or pneumonia (n = 7/9) [10, 14, 32, 35, 36, 38, 39], besides additional medical conditions such as diabetes, autoimmune diseases, cancer [39] or pregnancy [14]. One of the studies in the low-exposure group collected data related to the health condition of the selected subjects, but they did not report any specific exclusion criteria [37]. One study did not report any comorbidities or medical conditions of the subjects exposed to high PM levels [34].

## Discussion

This is the first systematic review of the influence of PM exposure level on the URT microbiome. Our results revealed that the URT microbiome composition and diversity are modified after exposure to high PM concentrations.

This systematic review summarized evidence for a total of 486 individuals from 9 different studies and revealed a slightly predominant negative association between microbiota diversity and exposure to PM for individuals with PM_2.5_ concentrations greater than 40 µg/m^3^ and the correspondingly elevated PM_10_ levels. Although the 40 µg/m^3^ threshold established in this systematic review is slightly higher than the threshold recommended by the WHO, even with looser thresholds, the effects are noticeable.

About microbial diversity, Mariani et al. [14] reported a positive association between microbiome diversity and PM in AR patients even at lower exposure levels, explained by the dysfunctional microbiota of those patients, which makes them more susceptible to microbiome alterations. Nevertheless, there was a slight predominant decrease in diversity among the reviewed studies after high PM exposure. We must consider that each individual study used their own criteria of “high” and “low” exposure, which led to different outcomes across the single studies.

In terms of microbiome composition, previous works have reported that Actinobacteria and Firmicutes are the predominant phyla in the nasopharynx microbiome of healthy individuals [40, 41], whereas in the oral cavity*,* the prevalence of Actinobacteria is lower than that in the nasopharynx, and Firmicutes is dominant, followed by Bacteroidetes and Proteobacteria [42]. In this systematic review, we identified a predominantly lower relative abundance of the Actinobacteria phylum among subjects exposed to high PM concentrations, but a clear dominance of this phylum in nasal swabs collected under low-PM conditions [14, 37]. In general, the most dominant phylum was Firmicutes, characterized by the production of spores, which make it resistant to harsh conditions and easily spreadable; however, microbial cells are more difficult to lyse, which leads to underrepresentation in metagenomic analysis [43]. This issue can explain the lack of a clear trend among the Italian studies related to Firmicutes phylum abundance. In contrast, the phylum Bacteroidetes was more represented in subjects exposed to high PM concentrations. The increase in Bacteroidetes is explained by its predominance in soils [44] which are carried on dust particles that are ultimately inhaled by humans. *Bacteroides* spp. are common commensal bacteria in the gut but can be opportunistic pathogens when displaced to different locations, such as the oral cavity, and are associated with infections such as pneumonia, chronic sinusitis and otitis, as well as lung and brain abscesses [45, 46]. Regarding Proteobacteria, a slight increase was observed associated with high PM exposure. Overgrowth of Proteobacteria has been previously associated with asthma [12]. In fact, in one of the included studies whose population was exposed to high PM levels, their subjects suffered from asthma and indeed presented high levels of Proteobacteria [10]. With respect to Cyanobacteria, its medium abundance was two times lower among low-exposure studies. This phylum has a low abundance (<0.08%) in healthy subjects [41], so the absence of data for Cyanobacteria in some of the reviewed studies is likely due to an insignificant contribution to the overall microbial abundance. The lack of differences observed at the phylum level between low and high exposure by Du et al. [36] may be due to the short period of exposure to low PM levels (less than a month), highlighting the long-lasting influence of short-term exposure to high airborne particles in the upper respiratory microbiome.

Nevertheless, we must also take into account the influence of the year season at the time of sampling. The most homogeneous microbiome composition was observed across studies conducted in fall, with an increase in Fusobacteria and Firmicutes, whereas Fusobacteria presented the lowest representation in spring. Our results partially agree with those of previous studies, that reported a predominance of Fusobacteria and Proteobacteria in children during fall [47]. However, Bogaert et al. (2011) described a predominance of Bacteroidetes and Firmicutes during spring, a trend not observed in this systematic review. In fact, two of the studies conducted during winter season [34, 39] reported the highest representation of Bacteroidetes, a phylum that increases the risk of opportunistic infections in the oral cavity [45, 46].

With respect to genus representation, most of the studies reported a positive association between PM exposure and *Moraxella* abundance, except for Zhao et al. [10], and a negative association with the commensal bacteria *Prevotella* spp. [10, 34, 39]. In the *Moraxella* genus, the common human respiratory tract pathogen *Moraxella catarrhalis* [48], together with *Haemophilus influenzae* and *Streptococcus pneumoniae, *are usually found in children with otitis media and sinusitis [49], but also in the oro- and nasopharyngeal microbiome of elderly individuals, with impact on their respiratory health [50]. High-pollution episodes such as smog periods can enrich the nasopharynx microbiome in these genera, as reported by Qin et al. [34], contributing to the development of severe respiratory diseases in populations at risk. Seasonal changes in the airborne microbiota promoted by meteorological fluctuations also modify the respiratory microbiome, with higher microbial diversity observed during winter [51], but also higher levels of airborne bacteria, such as *Streptococcus,* throughout this season [52]. This seasonal variation has also been associated with the exacerbation of asthma in both children and adults during fall and winter [53, 54]. Asthmatic patients were included in the study by Zhao et al. [10] during the winter, which must be considered a main factor influencing microbiome composition, together with the high PM_2.5_ exposure levels experienced by this population group.

The seasonality of the microbiome, which is associated with high PM exposure, leads to predominant colonization of the upper respiratory microbiome by pathogenic bacteria during winters, displacing beneficial microbes such as *Prevotella* [32, 34, 39], which has been proposed as a protective bacterium against *S. pneumoniae* [55]. Besides the microorganisms carried by PM, previous studies have reported a seasonality in the PM_2.5_ chemical composition [56], an alternative way in which PM_2.5_ may alter the airway epithelium, and afterwards its microbiome. Further studies in which both PM_2.5_ chemical and biological composition are determined, together with URT microbiome of people exposed, would be of value to further understand the mechanisms involved. Nevertheless, in general the composition of the microbiome is distributed more homogeneously by season than by PM exposure, indicating that season predetermines a particular microbiome composition. Because of that, seasonality must be considered as an influential factor of URT microbiome.

As reflected by Qin et al. [34], wearing masks is a fundamental barrier to avoid microbiome modifications caused by high pollution episodes, thus reducing the risk of respiratory infections. We must not forget the impact of the exposure to other environmental pollutants, such as NO_2_, which increases the abundances of mostly *Actinomyces*, *Actinomadura* and *Actinocorallia* (Actinobacteria phylum) and *Acinetobacter* (Proteobacteria phylum)[10]. Nevertheless, PM has been described as the major cause of respiratory health disorders among air pollutants [2, 57–59], so there is enough evidence to infer that the main alterations in the respiratory microbiome are due to PM exposure.

However, we should not ignore the geographical variation. All the studies were conducted in China except for two research studies by Mariani et al. [14, 37], which took place in Italy. This has led to potential bias in the results, as reflected by the different distribution of Actinobacteria and Bacteroidetes between countries, which may be confounded by geographical location, ethnicity and different genetic backgrounds of the participants involved, as well as by dietary patterns. For example, owing to the high prevalence of lactose intolerance in the Middle East, the Chinese diet does not include many dairy products [60]. There is wide knowledge on how diet can modulate the gut microbiome [61], but little is known about its influence on the respiratory flora. Daniele et al. [62] compared the salivary microbiota between vegan and Mediterranean diet patterns, and reported an enriched microbiome in people following a Mediterranean diet [62]. In addition, the gut-lung axis has been recently studied, revealing a potential link between the gut microbiome and lung diseases [63, 64]. These findings indicate that dietary patterns may influence the respiratory microbiome. Since both Italian studies belong to the low-exposure group, this must be considered a potential risk of bias in the present study.

In summary, the altered URT microbiome of people exposed to high PM levels reflected in this review may be the result of the colonization by new bacteria and the partial elimination of the host microbiome. Apart from direct microbiome alteration, previous studies have reported additional outcomes triggered by the inhalation of airborne particulates, that include the activation of proinflammatory responses [14, 35, 38]. Altogether, would increase the susceptibility of the population exposed to high levels of airway pollution to respiratory infections and allergies.

### Limitations

To correctly interpret the main outcomes obtained in this systematic review, several limitations across the studies should be considered. First, exposure assessment devices differed among studies, which implies that PM measurements have non-heterogeneous deviations. As stated in the European air quality regulation, the measurement of PM_2.5_ levels with reference gravimetric systems has a legal maximum uncertainty of 25% at the limit value. Since the European equipment used for PM measurement must adhere to the mentioned regulations and it is subjected to regular intercomparisons with US and Chinese equipment, the PM concentration data included in this review can be considered equivalent. Second, the lack of publicly available raw 16S rRNA sequencing data and the different software and reference databases used for the analysis of 16S rRNA sequences across the studies, along with the lack of full information about the parameters employed during the data trimming and filtering process, make it impossible to perform a proper statistical analysis of the microbiome data. Third, this revision identified an important shortage of confounder control in most of the studies, which has been considered a risk of bias in the present systematic review. First, air pollution includes not only PM but also O_3_, NO, NO_2_, CO, and SO_2_, which should be taken into consideration. Additionally, inhalation of cigar smoke and antibiotic use have important impacts on the respiratory tract microbiome [65], but not all studies have considered these factors as exclusion criteria for their study population. Additionally, asthma patients were included in one study, so those differences in population characteristics constitute a limitation of the present systematic review. Finally, different sample types were included in the selected studies, although only unstimulated saliva or swab samples were analyzed to reduce sampling bias [66]. There is a lack of studies conducting microbiome analysis on lower respiratory tract locations, such as sputum samples, which precluded its inclusion on this systematic review. All these limitations must be taken into consideration as important biases for the comparison of the main findings of the selected studies and highlight the necessity of conducting further research on the respiratory microbiome with a well-established study design that considers all potential confounders.

### Recommendations

It would be of great interest, as guidance for the proper study of the upper respiratory microbiome and on the basis of the STORMS quality guidance, that the following considerations should be taken into account: 16S rRNA sequencing studies should upload their raw data sequences on open databases, as a claim for science data transparency to conduct accurate meta-analysis; detailed workflow of the sequencing process (sample shipping and storage time and conditions, RNA extraction and amplification protocols specifying the 16S rRNA target region, and parameter settings during 16S rRNA sequence trimming, filtering and data normalization, which can be provided as open source code) should be reported; and finally, all potential confounders should be controlled throughout the study, which includes additional pollutants, antibiotic use, smoking, or any respiratory-related disease conditions of the individuals participating in the study.

With further and well conducted research, we will overcome the shortage of respiratory microbiome studies that consider PM exposure, reflected in the low number of studies included in this systematic review (n = 9).

## Conclusion

In conclusion, exposure to highly polluted urban areas with elevated PM levels has a direct effect on the upper respiratory tract microbiome balance, which may have associated health consequences such as inflammatory respiratory disorders like allergic rhinitis, rhinosinusitis, or severe respiratory infections.

## Supplementary Information

Additional file 1: Assessment of the quality of selected studies following the “Strengthening the Organization and Reporting of Microbiome Studies” checklist

Additional file 2: Summary of the DNA extraction methods and sequencing processing pipelines used in each study. When available, the version of the software used is indicated

Additional file 3: Statistically significant variations on alpha-diversity after exposure to high PM levels reported by the reviewed studies

Additional file 4: Differences in the distribution of the upper respiratory microbiome relative abundance at the phylum level by author, grouped in high or low PM10 exposure, and by phylum level among high and low PM_10_ exposure groups

Additional file 5: Differences in the distribution of the upper respiratory microbiome composition by phylum among countries. The data are presented as the relative abundances estimated from raw data or reported by the individual studies, for each phylum

Additional file 6: Estimated quality of the selected studies, according to the criteria established by the Collaboration for Environmental Evidence Critical Appraisal Tool

## Data Availability

The authors declare that the data supporting the findings of this study are available within the paper and its Supplementary Information files (Additional files 1–6). Should any raw data files be needed in another format they are available from the corresponding author upon reasonable request.
