# Supplementary material for: Effects of urban airborne particulate matter exposure on the human upper respiratory tract microbiome: a systematic review
Source: Respir Res. 2025 Mar 28;26:118. doi: 10.1186/s12931-025-03179-9 (PMC11954284; doi:10.1186/s12931-025-03179-9)
Supplement: Supplementary file 1 — Additional file 1: Assessment of the quality of selected studies following the “Strengthening the Organization and Reporting of Microbiome Studies” checklist [file 12931_2025_3179_MOESM1_ESM.docx]

**Additional file 1**. Assessment of the quality of selected studies following the “Strengthening the Organization and Reporting of Microbiome Studies” (STORMS) checklist.

| **Number** | **Item** | **Recommendation** | **Mariani et al (2018)** | **Mariani et al (2021)** | | **Lin et al (2022)** | | **Li et al (2019)** | | **Qin et al (2019)** | | **Zhao et al (2020a)** | | **Zhao et al (2020b)** | | **Du et al (2023)** | | **Li et al (2023a)** |  |
| --- | --- | --- | --- | --- | --- | --- | --- | --- | --- | --- | --- | --- | --- | --- | --- | --- | --- | --- | --- |
| **ABSTRACT** | |  |  | |  | |  | |  | |  | |  | |  | |  | | |
| 1.0 | Structured or Unstructured Abstract | Abstract should include information on background, methods, results, and conclusions in structured or unstructured format. | Yes | Yes | | Yes | | Yes | | Yes | | Yes | | Yes | | Yes | | Yes |  |
| 1.1 | Study Design | State study design in abstract. | Yes | Yes | | Yes | | Yes | | Yes | | Yes | | Yes | | Yes | | Yes |  |
| 1.2 | Sequencing methods | State the strategy used for metagenomic classification. | Yes | Yes | | Yes | | Yes | | Yes | | Yes | | Yes | | Yes | | Yes |  |
| 1.3 | Specimens | Describe body site(s) studied. | Yes | Yes | | Yes | | Yes | | Yes | | Yes | | Yes | | Yes | | Yes |  |
| **INTRODUCTION** | |  |  | |  | |  | |  | |  | |  | |  | |  | | |
| 2.0 | Background and Rationale | Summarize the underlying background, scientific evidence, or theory driving the current hypothesis as well as the study objectives. | Yes | Yes | | Yes | | Yes | | Yes | | Yes | | Yes | | Yes | | Yes |  |
| 2.1 | Hypotheses | State the pre-specified hypothesis. If the study is exploratory, state any pre-specified study objectives. | Yes | Yes | | Yes | | Yes | | Yes | | Yes | | Yes | | Yes | | Yes |  |
| **METHODS** | |  |  | |  | |  | |  | |  | |  | |  | |  | | |
| 3.0 | Study Design | Describe the study design. | Yes | Yes | | Yes | | Yes | | Yes | | Yes | | Yes | | Yes | | Yes |  |
| 3.1 | Participants | State what the population of interest is, and the method by which participants are sampled from that population. Include relevant information on physiological state of the subjects or stage in the life history of disease under study when participants were sampled. | Yes | Yes | | Yes | | Yes | | Yes | | Yes | | Yes | | Yes | | Yes |  |
| 3.2 | Geographic location | State the geographic region(s) where participants were sampled from. | Yes | Yes | | Yes | | Yes | | Yes | | Yes | | Yes | | Yes | | Yes |  |
| 3.3 | Relevant Dates | State the start and end dates for recruitment, follow-up, and data collection. | Yes | Yes | | Yes | | Yes | | Yes | | Yes | | Yes | | Yes | | Yes |  |
| 3.4 | Eligibility criteria | List any criteria for inclusion and exclusion of recruited participants. | Yes | Yes | | Yes | | Yes | | Yes | | Yes | | Yes | | Yes | | Yes |  |
| 3.5 | Antibiotics Usage | List what is known about antibiotics usage before or during sample collection. | No | No | | No | | Yes | | Yes | | Yes | | Yes | | Yes | | Yes |  |
| 3.6 | Analytic sample size | Explain how the final analytic sample size was calculated, including the number of cases and controls if relevant, and reasons for dropout at each stage of the study. This should include the number of individuals in whom microbiome sequencing was attempted and the number in whom microbiome sequencing was successful. | Yes | Yes | | Yes | | Yes | | Yes | | Yes | | Yes | | Yes | | Yes |  |
| 3.7 | Longitudinal Studies | For longitudinal studies, state how many follow-ups were conducted, describe sample size at follow-up by group or condition, and discuss any loss to follow-up. | NA | NA | | Yes | | NA | | Yes | | Yes | | NA | | Yes | | Yes |  |
| 3.8 | Matching | For matched studies, give matching criteria. |  |  | |  | |  | |  | |  | |  | |  | |  |  |
| 3.9 | Ethics | State the name of the institutional review board that approved the study and protocols, protocol number and date of approval, and procedures for obtaining informed consent from participants. | Yes | Yes | | Yes | | No | | yes | | Yes | | Yes | | Yes | | Yes |  |
| 4.0 | Laboratory methods | State the laboratory/center where laboratory work was done. | Yes | Yes | | Yes | | Yes | | Yes | | Yes | | Yes | | Yes | | Yes |  |
| 4.1 | Specimen collection | State the body site(s) sampled from and how specimens were collected. | Yes | Yes | | Yes | | Yes | | Yes | | Yes | | Yes | | Yes | | Yes |  |
| 4.2 | Shipping | Describe how samples were stored and shipped to the laboratory. | No | No | | No | | Yes | | Yes | | Yes | | Yes | | Yes | | No |  |
| 4.3 | Storage | Describe how the laboratory stored samples, including time between collection and storage and any preservation buffers or refrigeration used. | No | Yes | | No | | Yes | | No | | Yes | | Yes | | Yes | | No |  |
| 4.4 | DNA extraction | Provide DNA extraction method, including kit and version if relevant. | Yes | Yes | | Yes | | Yes | | Yes | | Yes | | Yes | | Yes | | Yes |  |
| 4.5 | Human DNA sequence depletion or microbial DNA enrichment | Describe whether human DNA sequence depletion or enrichment of microbial or viral DNA was performed. | No | No | | No | | No | | No | | No | | No | | No | | No |  |
| 4.6 | Primer selection | Provide primer selection and DNA amplification methods as well as variable region sequenced (if applicable). | Yes | Yes | | Yes | | Yes | | Yes | | Yes | | Yes | | Yes | | Yes |  |
| 4.7 | Positive Controls | Describe any positive controls (mock communities) if used. | No | No | | No | | No | | No | | No | | No | | No | | No |  |
| 4.8 | Negative Controls | Describe any negative controls if used. | No | No | | No | | No | | No | | No | | No | | Yes | | No |  |
| 4.9 | Contaminant mitigation and identification | Provide any laboratory or computational methods used to control for or identify microbiome contamination from the environment, reagents, or laboratory. | No | No | | No | | No | | No | | No | | No | | Yes | | No |  |
| 4.10 | Replication | Describe any biological or technical replicates included in the sequencing, including which steps were replicated between them. | No | No | | No | | No | | No | | No | | No | | No | | No |  |
| 4.11 | Sequencing strategy | Major divisions of strategy, such as shotgun or amplicon sequencing. | Yes | Yes | | Yes | | Yes | | Yes | | Yes | | Yes | | Yes | | Yes |  |
| 4.12 | Sequencing methods | State whether experimental quantification was used (QMP/cell count based, spike-in based) or whether relative abundance methods were applied. | Yes | Yes | | No | | Yes | | Yes | | Yes | | Yes | | Yes | | Yes |  |
| 4.13 | Batch effects | Detail any blocking or randomization used in study design to avoid confounding of batches with exposures or outcomes. Discuss any likely sources of batch effects, if known. | No | No | | No | | Yes | | No | | No | | No | | No | | No |  |
| 4.14 | Metatranscriptomics | Detail whether any mRNA enrichment was performed and whether/how retrotranscription was performed prior to sequencing. Provide size range of isolated transcripts. Describe whether the sequencing library was stranded or not. Provide details on sequencing methods and platforms. | NA | NA | | NA | | NA | | NA | | NA | | NA | | NA | | NA |  |
| 4.15 | Metaproteomics | Detail which protease was used for digestion. Provide details on proteomic methods and platforms. | NA | NA | | NA | | NA | | NA | | NA | | NA | | NA | | NA |  |
| 4.16 | Metabolomics | Specify the analytic method used. For mass spectrometry, detail which fractions were obtained and how these were analyzed. Provide details on metabolomics methods and platforms. | NA | NA | | NA | | NA | | NA | | NA | | NA | | NA | | NA |  |
| 5.0 | Data sources/ measurement | For each non-microbiome variable, including the health condition, intervention, or other variable of interest, state how it was defined, how it was measured or collected, and any transformations applied to the variable prior to analysis. | NA | Yes | | Yes | | Yes | | NA | | Yes | | Yes | | Yes | | Yes |  |
| 6.0 | Research design for causal inference | Discuss any potential for confounding by variables that may influence both the outcome and exposure of interest. State any variables controlled for and the rationale for controlling for them. | No | No | | Yes | | Yes | | No | | Yes | | Yes | | Yes | | Yes |  |
| 6.1 | Selection bias | Discuss potential for selection or survival bias. | NA | NA | | NA | | NA | | NA | | NA | | NA | | NA | | NA |  |
| 7.0 | Bioinformatic and Statistical Methods | Describe any transformations to quantitative variables used in analyses (e.g. use of percentages instead of counts, normalization, rarefaction, categorization). | Yes | Yes | | Yes | | No | | No | | Yes | | Yes | | Yes | | Yes |  |
| 7.1 | Quality Control | Describe any methods to identify or filter low quality reads or samples. | Yes | Yes | | Yes | | Yes | | Yes | | Yes | | Yes | | Yes | | Yes |  |
| 7.2 | Sequence analysis | Describe any taxonomic, functional profiling, or other sequence analysis performed. | Yes | Yes | | Yes | | Yes | | Yes | | Yes | | Yes | | Yes | | Yes |  |
| 7.3 | Statistical methods | Describe all statistical methods. | Yes | Yes | | Yes | | Yes | | Yes | | Yes | | Yes | | Yes | | Yes |  |
| 7.4 | Longitudinal analysis | If the study is longitudinal, include a section that explicitly states what analysis methods were used (if any) to account for grouping of measurements by individual or patterns over time. | NA | NA | | No | | No | | Yes | | Yes | | NA | | Yes | | Yes |  |
| 7.5 | Subgroup analysis | Describe any methods used to examine subgroups and interactions. | NA | NA | | NA | | NA | | NA | | NA | | NA | | NA | | NA |  |
| 7.6 | Missing data | Explain how missing data were addressed. | NA | NA | | NA | | NA | | NA | | NA | | NA | | Yes | | Yes |  |
| 7.7 | Sensitivity analyses | Describe any sensitivity analyses. | No | No | | No | | No | | No | | No | | No | | No | | No |  |
| 7.8 | Findings | State criteria used to select findings for reporting. | Yes | Yes | | Yes | | Yes | | Yes | | Yes | | Yes | | Yes | | Yes |  |
| 7.9 | Software | Cite all software (including read mapping software) and databases (including any used for taxonomic reference or annotating amplicons, if applicable) used. Include version numbers. | Yes | Yes | | Cite versions, but not packages or parameters | | Cites versions and packages, but not parameters | | Cite versions, but not packages or parameters | | Yes | |  |  |  |  |  |  |
| 8.0 | Reproducible research | Make a statement about whether and how others can reproduce the reported analysis. | No | Yes | | No | | No | | No | | No | | No | | Yes | | No |  |
| 8.1 | Raw data access | State where raw data may be accessed including demultiplexing information. | No | Yes | | No | | No | | No | | No | | No | | Yes | | No |  |
| 8.2 | Processed data access | State where processed data may be accessed. | No | Yes | | No | | No | | No | | No | | No | | Yes | | No |  |
| 8.3 | Participant data access | State where individual participant data such as demographics and other covariates may be accessed, and how they can be matched to the microbiome data. | No | No | | No | | No | | No | | No | | No | | Yes | | No |  |
| 8.4 | Source code access | State where code may be accessed. | No | No | | No | | No | | No | | No | | No | | Yes | | No |  |
| 8.5 | Full results | Provide full results of all analyses, in computer-readable format, in supplementary materials. | No | Yes, but not in plain-text format | | No | | No | | Yes | | Yes | | Yes, but not in plain-text format | | Yes | | No |  |
| **RESULTS** | |  |  | |  | |  | |  | |  | |  | |  | |  | | |
| 9.0 | Descriptive data | Give characteristics of study participants and information on exposures and potential confounders. | Yes | Yes | | Yes | | Yes | | Yes | | Yes | | Yes | | Yes | | Yes |  |
| 10.0 | Microbiome data | Report descriptive findings for microbiome analyses with all applicable outcomes and covariates. | Yes | Yes | | Yes | | No | | Yes | | Yes | | Yes | | Yes | | No |  |
| 10.1 | Taxonomy | Identify taxonomy using standardized taxon classifications that are sufficient to uniquely identify taxa. | Yes | Yes | | Yes, in graphic format | | Yes | | Yes | | Yes | | Yes | | Yes | | Yes |  |
| 10.2 | Differential abundance | Report results of differential abundance analysis by the variable of interest and (if applicable) by time, clearly indicating the direction of change and total number of taxa tested. | Yes | Yes | | No | | Yes | | Yes | | Yes | | Yes | | Yes | | Yes |  |
| 10.3 | Other data types | Report other data analyzed. | NA | NA | | Yes, KEGG mathways | | NA | | NA | | NA | | Yes, metabolomic | | NA | | Yes |  |
| 10.4 | Other statistical analysis | Report any statistical data analysis not covered above. | NA | NA | | NA | | NA | | NA | | NA | | NA | | NA | | NA |  |
| **DISCUSSION** | |  |  | |  | |  | |  | |  | |  | |  | |  | | |
| 11.0 | Key results | Summarise key results with reference to study objectives | Yes | Yes | | Yes | | Yes | | Yes | | Yes | | Yes | | Yes | | Yes |  |
| 12.0 | Interpretation | Give a cautious overall interpretation of results considering objectives, limitations, multiplicity of analyses, results from similar studies, and other relevant evidence. | Yes | Yes | | Yes | | Yes | | Yes | | Yes | | Yes | | Yes | | Yes |  |
| 13.0 | Limitations | Discuss limitations of the study, taking into account sources of potential bias or imprecision. | Yes | Yes | | Yes | | Yes | | Yes | | No | | Yes | | Yes | | Yes |  |
| 13.1 | Bias | Discuss any potential for bias to influence study findings. | Yes | Yes | | Yes | | No | | Yes | | No | | Yes | | Yes | | Yes |  |
| 13.2 | Generalizability | Discuss the generalisability (external validity) of the study results | Yes | Yes | | Yes | | Yes | | Yes | | No | | Yes | | Yes | | Yes |  |
| 14.0 | Ongoing/future work | Describe potential future research or ongoing research based on the study's findings. | Yes | No | | Yes | | Yes | | No | | Yes | | Yes | | Yes | | Yes |  |
| **OTHER INFORMATION** | |  |  | |  | |  | |  | |  | |  | |  | |  | | |
| 15.0 | Funding | Give the source of funding and the role of the funders for the present study and, if applicable, for the original study on which the present article is based | Yes | Yes | | Yes | | Yes | | Yes | | Yes | | Yes | | Yes | | Yes |  |
| 15.1 | Acknowledgements | Include acknowledgements of those who contributed to the research but did not meet critera for authorship. | Yes | Yes | | Yes | | No | | Yes | | Yes | | Yes | | Yes | | NA |  |
| 15.2 | Conflicts of Interest | Include a conflicts of interest statement. | No | Yes | | No | | No | | Yes | | Yes | | Yes | | Yes | | Yes |  |
| 16.0 | Supplements | Indicate where supplements may be accessed and what materials they contain. | Yes | Yes | | Yes | | Yes | | Yes | | Yes | | Yes | | Yes | | Yes |  |
| 17.0 | Supplementary data | Provide supplementary data files of results with for all taxa and all outcome variables analyzed. Indicate the taxonomic level of all taxa. | Yes | Yes, for genera level | | No | | No | | Yes | | Yes | | Yes, in graphic format | | Yes | | No |  |
