# Supplementary material for: Effects of urban airborne particulate matter exposure on the human upper respiratory tract microbiome: a systematic review
Source: Respir Res. 2025 Mar 28;26:118. doi: 10.1186/s12931-025-03179-9 (PMC11954284; doi:10.1186/s12931-025-03179-9)
Supplement: Supplementary file 2 — Additional file 2: Summary of the DNA extraction methods and sequencing processing pipelines used in each study. When available, the version of the software used is indicated [file 12931_2025_3179_MOESM2_ESM.docx]

**Additional file 2.** Summary of the DNA extraction methods and sequencing processing pipelines used in each study. When available, the version of the software used is indicated.

| Author | DNA extraction | Sequencing platform | Upstream analyses | Chimeric | OTU clustering algorithm | Taxonomic database | Taxonomic classification tool |
| --- | --- | --- | --- | --- | --- | --- | --- |
| Mariani et al (2018) | Diam® UCP Pathogen Mini | Illumina MiSeq | Trimmomatic v0.32 + Fastq-join.py tool + QIIME 1.9.1 |  | USEARCH61 | Greengenes v13.8 | RDP classifier |
| Mariani et al (2021) | QIAamp® UCP Pathogen Mini | Illumina MiSeq | Trimmomatic v0.32 + Fastq-join.py tool + QIIME 1.9.1 | USEARCH61 | USEARCH61 | Greengenes v13.8 | RDP classifier |
| Lin et al (2022) | MN NuceloSpin 96 Soil kit | illumina NovaSeq6000 | Trimmomatic v0.33 & Cutadapt v1.9.1 | UCHIME v4.2 | USEARCH v10 | Silva database | RDP classifier |
| Li et al (2019) | UltraClean® Tissue & Cells DNA Isolation Kit (Qiagen) | Illumina MiSeq | QIIME 1.9.1 | UCHIME in USEARCH v8.1.1861 | Uclust | Mothur & Greengenes | QIIME |
| Qin et al (2019) | QIAamp DNA Mini kit (Qiagen) | Illumina HiSeq | QIIME | UCHIME algorithm | Uparse v7.0.1001 | SILVA v123 | RDP classifier |
| Zhao et al (2020a) | QIAamp DNA Mini Kit (Qiagen) | Illumina MiSeq | QIIME v1.8.0 |  | UCLUST | Greengenes v13.8 | BLAST |
| Zhao et al (2020b) | QIAamp DNA Mini Kit (Qiagen) | Illumina Miseq | FLASH & QIIME v1.8.0 | USEARCH v5.2.236 | UCLUST | Greengenes | BLAST |
| Du et al (2023) | Maxwell RSC whole blood DNA kit (Promega) | Illumina MiSeq | VSEARCH v2.7.1 |  | UNOISE3 | SILVA v123 | SINTAX |
| Li et al (2023a) | Cetyltrimethyl Ammonium Bromide/Sodium Dodecyl Sulfonate method | Ion S5™ XL platform | Cutadapt & QIIME 1.9.1 |  |  | Greengenes v13.8 |  |
