# Supplementary material for: Effects of urban airborne particulate matter exposure on the human upper respiratory tract microbiome: a systematic review
Source: Respir Res. 2025 Mar 28;26:118. doi: 10.1186/s12931-025-03179-9 (PMC11954284; doi:10.1186/s12931-025-03179-9)
Supplement: Supplementary file 3 — Additional file 3: Statistically significant variations on alpha-diversity after exposure to high PM levels reported by the reviewed studies [file 12931_2025_3179_MOESM3_ESM.pdf]

Additional file 3: Statistically significant variations on alpha-diversity after exposure to high PM levels reported by the reviewed studies.

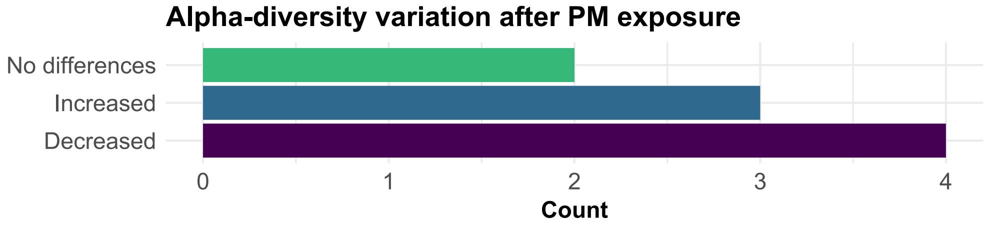

Note: The study of Mariani et al (2021) is depicted twice, since they reported a significant increase in alpha-diversity in individuals with allergic rhinitis, but a significant decrease in healthy subjects.
