# Supplementary material for: Effects of urban airborne particulate matter exposure on the human upper respiratory tract microbiome: a systematic review
Source: Respir Res. 2025 Mar 28;26:118. doi: 10.1186/s12931-025-03179-9 (PMC11954284; doi:10.1186/s12931-025-03179-9)
Supplement: Supplementary file 4 — Additional file 4: Differences in the distribution of the upper respiratory microbiome relative abundance at the phylum level by author, grouped in high or low PM10 exposure, and by phylum level among high and low PM10 exposure groups [file 12931_2025_3179_MOESM4_ESM.pdf]

Additional file 4. Differences in the distribution of the upper respiratory microbiome relative abundance at the phylum level by author, grouped in high or low PM_{10} exposure (**a**), and by phylum level among high and low PM_{10} exposure groups (**b**).

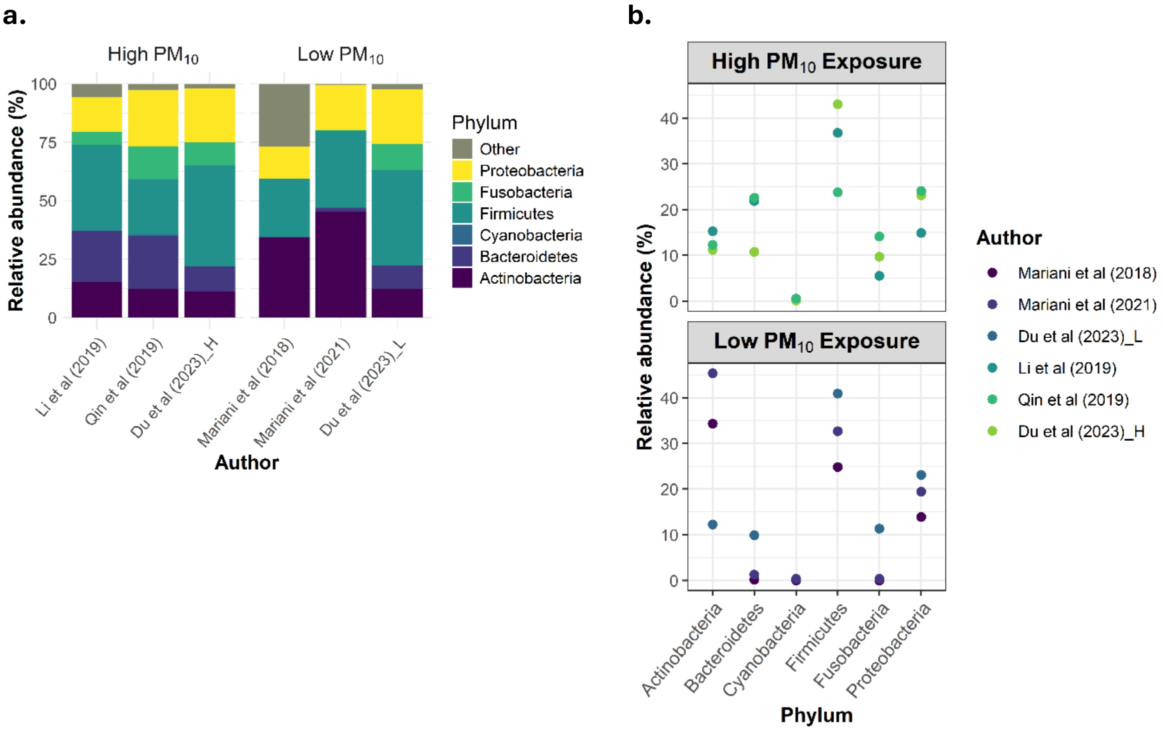

Note: We have established the PM_{10} cutoff based on the evidence that $PM_{10}/PM_{2.5}$ concentration ratios are usually in the range of 1.5-2 [1, 2]. Then, for a $PM_{2.5}$ cutoff of 40 µg/mL and considering a minimum ratio of 1.5, the corresponding PM_{10} cutoff is 60 µg/mL

1. Munir S. Analysing Temporal Trends in the Ratios of PM2.5/PM10 in the UK. Aerosol Air Qual Res. 2017;17:34-48.
2. Zha H, Wang R, Feng X, An C, Qian J. Spatial characteristics of the PM2.5/PM10 ratio and its indicative significance regarding air pollution in Hebei Province, China. Environmental Monitoring and Assessment. 2021;193:1-12.
