# Supplementary material for: Effects of urban airborne particulate matter exposure on the human upper respiratory tract microbiome: a systematic review
Source: Respir Res. 2025 Mar 28;26:118. doi: 10.1186/s12931-025-03179-9 (PMC11954284; doi:10.1186/s12931-025-03179-9)
Supplement: Supplementary file 5 — Additional file 5: Differences in the distribution of the upper respiratory microbiome composition by phylum among countries. The data are presented as the relative abundances estimated from raw data or reported by the individual studies, for each phylum [file 12931_2025_3179_MOESM5_ESM.pdf]

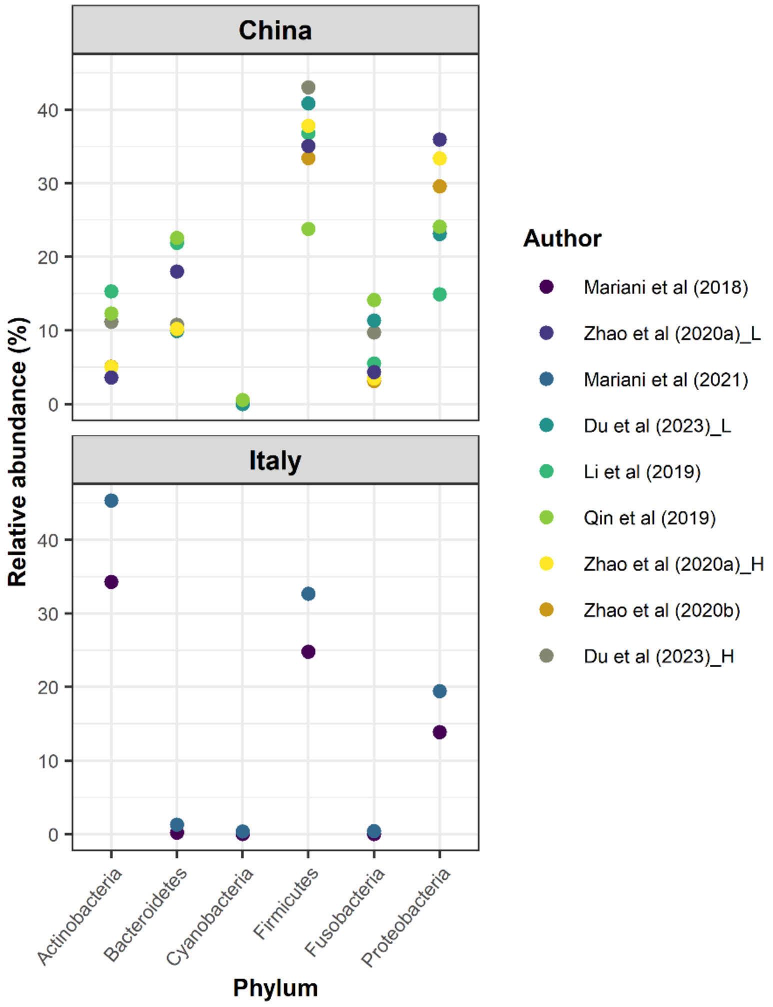

Additional file 5. Differences in the distribution of the upper respiratory microbiome composition by phylum among countries (China and Italy). The data are presented as the relative abundances (%) estimated from raw data (Mariani et al, 2021, and Du et al, 2023) or reported by the individual studies (rest), for each phylum.
