# Supplementary material for: Effects of urban airborne particulate matter exposure on the human upper respiratory tract microbiome: a systematic review
Source: Respir Res. 2025 Mar 28;26:118. doi: 10.1186/s12931-025-03179-9 (PMC11954284; doi:10.1186/s12931-025-03179-9)
Supplement: Supplementary file 6 — Additional file 6: Estimated quality of the selected studies, according to the criteria established by the Collaboration for Environmental Evidence Critical Appraisal Tool [file 12931_2025_3179_MOESM6_ESM.docx]

**Additional file 6.** Estimated quality of the selected studies, according to the criteria established by the Collaboration for Environmental Evidence Critical Appraisal Tool (CEECAT).

| **Nº** | **Criterion** | **Mariani et al (2018)** | **Mariani et al (2021)** | **Lin et al (2022)** | **Li et al (2019)** | **Qin et al (2019)** | **Zhao et al (2020a)** | **Zhao et al (2020b)** | **Du et al (2023)** | **Li et al (2023)** |
| --- | --- | --- | --- | --- | --- | --- | --- | --- | --- | --- |
| **1** | **Risk of confounding biases** | High | High | Medium | Low | High | Low | Low | Medium | Low |
| **2** | **Risk of post-intervention/exposure selection biases** | Medium | Low | Medium | Low | Low | Low | Low | Medium | Medium |
| **3** | **Risk of misclassified comparison biases** | Medium | Low | Low | Low | Low | Low | Low | Low | Low |
| **4** | **Risk of performance biases** | NA | NA | NA | NA | NA | NA | NA | NA | NA |
| **5** | **Risk of detection biases** | Low | Low | Low | Low | Low | Low | Low | Low | Low |
| **6** | **Risk of outcome reporting biases** | Low | Low | Low | Low | Low | Low | Low | Low | Low |
| **7** | **Risk of outcome assessment biases** | Medium | Medium | Medium | Medium | Medium | Medium | Medium | Low | Medium |
|  | **OVERALL** | **High** | **High** | **Medium** | **Medium** | **High** | **Medium** | **Medium** | **Medium** | **Medium** |
